# Proteostasis and Mitochondrial Role on Psychiatric and Neurodegenerative Disorders: Current Perspectives

**DOI:** 10.1155/2018/6798712

**Published:** 2018-06-27

**Authors:** Pablo Olivero, Carlo Lozano, Ramón Sotomayor-Zárate, Nicolás Meza-Concha, Marcelo Arancibia, Claudio Córdova, Wilfredo González-Arriagada, Ricardo Ramírez-Barrantes, Ivanny Marchant

**Affiliations:** ^1^Laboratorio de Estructura y Función Celular, Escuela de Medicina, Facultad de Medicina, Universidad de Valparaíso, Hontaneda 2664, 2341386 Valparaíso, Chile; ^2^Centro Interoperativo en Ciencias Odontológicas y Médicas, Universidad de Valparaíso, Valparaíso, Chile; ^3^Servicio de Anatomía Patológica, Hospital Carlos van Buren, San Ignacio 725, Valparaíso, Chile; ^4^Laboratorio de Neuroquímica y Neurofarmacología, Centro de Neurobiología y Fisiopatología Integrativa, Instituto de Fisiología, Facultad de Ciencias, Universidad de Valparaíso, Valparaíso, Chile; ^5^Patología y Diagnóstico Oral, Facultad de Odontología, Universidad de Valparaíso, Valparaíso, Chile; ^6^Escuela de Tecnologia Medica, Universidad Andres Bello, Quillota 980, 2531015 Viña del Mar, Chile

## Abstract

Proteostasis involves processes that are fundamental for neural viability. Thus, protein misfolding and the formation of toxic aggregates at neural level, secondary to dysregulation of the conservative mechanisms of proteostasis, are associated with several neuropsychiatric conditions. It has been observed that impaired mitochondrial function due to a dysregulated proteostasis control system, that is, ubiquitin-proteasome system and chaperones, could also have effects on neurodegenerative disorders. We aimed to critically analyze the available findings regarding the neurobiological implications of proteostasis on the development of neurodegenerative and psychiatric diseases, considering the mitochondrial role. Proteostasis alterations in the prefrontal cortex implicate proteome instability and accumulation of misfolded proteins. Altered mitochondrial dynamics, especially in proteostasis processes, could impede the normal compensatory mechanisms against cell damage. Thereby, altered mitochondrial functions on regulatory modulation of dendritic development, neuroinflammation, and respiratory function may underlie the development of some psychiatric conditions, such as schizophrenia, being influenced by a genetic background. It is expected that with the increasing evidence about proteostasis in neuropsychiatric disorders, new therapeutic alternatives will emerge.

## 1. Introduction

Ramón y Cajal, a pioneer in neuroscience, was the first to describe neurons as brain units that compose “cellular societies,” from the point of view of functional morphology [1]. The story continues at the *Université de Paris*, where doctor Jean-Martin Charcot creates a chair on which all modern neurobiology develops. In fact, the autopsies performed by Charcot in illegitimate prostitutes' sons at the *Hôpital de la Salpêtrière* would change the vision of emerging neurobiology forever. Thanks to his contribution, it was possible to determine the existence of certain neuromuscular diseases and rudimentarily identify pathologies such as multiple sclerosis and Parkinson's disease. One hundred sixty years later, neuropathology could contribute to the study of neurodegenerative disorders through conventional techniques, for example, histopathology, histochemistry, or immunohistochemistry applied to the analysis of changes in normal distribution of various types of proteins in neurons and tissues. Then, in the 90s, the presence of the so-called inclusion bodies was demonstrated in prevalent neurodegenerative conditions like Alzheimer's disease (AD), Parkinson's disease (PD), Huntington's disease (HD), amyotrophic lateral sclerosis, polyglutamine diseases, and the Lewy body dementia. At the same time, immunohistochemistry revealed the role of the ubiquitin-proteasome system and molecular chaperones in the formation of inclusion bodies, particularly in AD and PD [2–7]. Nowadays, we know that besides neuronal involution and reactive gliosis, most neurodegenerative diseases are characterized by protein accumulation.

In protein biosynthesis, metabolic changes, mutations, and stress are frequent conditions that cause protein misfolding and hamper proper biological function. Being molecular machineries whose constituent elements are chaperones, the ubiquitin-proteasome system and the autophagy-lysosomal system [8, 9] constantly counteract these risks by avoiding the accumulation of nondegradable protein aggregates and the consequent cellular malfunction and death [10]. Preserving proteostasis, that is, stable conditions during processes such as biogenesis, folding, trafficking, or degradation of proteins, is crucial to guaranteeing cell functions and the ability to elaborate pertinent reactions to tissue-specific chronic and acute stressors [11]. Dysregulation of the conservative mechanisms of proteostasis involves processes that are fundamental for the viability of postmitotic cells such as neurons and has been associated with several neurodegenerative diseases, such as AD, PD, and HD, among others [12]. Thereby, with increasing knowledge about changes in the tissue protein distribution, new pathogenic mechanisms could be revealed as potential therapeutic targets, especially in the study of the ubiquitin-proteasome system and molecular chaperones [8, 13]. Besides, it has been observed that impaired mitochondrial function, which is influenced by the ubiquitin-proteasome system and chaperones, may also have effects on neurodegenerative disorders [14]. In the present work, we will review the main findings on the neurobiological implications of proteostasis, from a molecular perspective, in relation to the development of neurodegenerative and neuropsychiatric diseases. We discuss the genetic and molecular considerations of mitochondrial dysfunction, an important organelle in proteostasis, in schizophrenia. We conducted an exhaustive bibliographic search through the available articles on MEDLINE/PubMed database. Here, we present the main findings of the available literature, focusing on three main topics: (1) proteostasis in neurodegenerative disease, (2) TRPV1 and proteostasis, and (3) proteostasis and mitochondrial dysfunction in schizophrenia.

### 1.1. Proteostasis in Neurodegenerative Disease

The efficiency of the cellular physiological processes depends on proper protein localization and function. There is a molecular network that participates in the intricate mechanisms of synthesis, folding, trafficking, and degradation necessary to ensure the structure and function of proteins [15]. The maintenance of proteostasis thus involves the translational and folding machinery including their regulatory systems such as the unfolded protein response (UPR), as well as the large group of molecular chaperones such as HSP70, HSP90, DNAJ/HSP40, chaperonin/HSP60, and small HSP (sHSP) families [16, 17], which balance protein function and turnover. Chaperones' ATP-dependent function is impaired in cellular stress condition. Thus, poor physical recognition by chaperone networks and cellular metabolic stress condition may contribute to protein aggregation in aging and disease [18]. Proteins can be degraded individually or massively mainly in proteasomes by the ubiquitin-proteasome system (UPS) [15]. The UPS is part of the extensive system for protein quality control of neurons and other types of cells, regulating the degradation of misfolding or aberrant proteins to prevent detrimental aggregation. The proteins that will be degraded by the UPS are first ubiquitylated via a series of enzymatic reactions involving ubiquitin-activating (E1), conjugation (E2), and ligase (E3) enzymes [15]. Proteasomes include two subcomplexes, the core particle (CP, 20S) which is a barrel-shaped structure composed of four stacked rings, two identical outer *α* rings and two identical inner *β* rings, which carry the catalytic activity, and the regulatory particle (19S) which caps the extremities of the barrel and regulates the entry of ubiquitylated proteins into the catalytic center. The proteasomal network composition is highly dynamic; the levels of molecular chaperones and proteasome subunits can increase or decrease globally or specifically in some compartment, depending on factors such as environmental changes, genetic factors, and aging phenomena [19]. These changes lessen the ability of cells to regulate the accumulation of misfolded proteins, which can induce cell dysfunction and death [15]. The UPS in particular is able to modulate synaptic physiology both pre- and postsynaptically. UPS participation in the neuronal synapse implies regulating calcium channels and may have an impact on long-term memory [20].

Dysfunction of the proteostatic network decreases neuronal plasticity [21–23]. It has been reported that in response to LTP-inducing stimuli in the hippocampus, the activity of the proteasomes increases and also after activation of NMDAR [19]. The kinase CaMKII*α* is activated by an entry of calcium via NMDAR which in turn phosphorylates and enhances the proteolytic activity of the proteasome, coupling the synaptic excitation with changes in proteostasis [19].

In mammalian neurons, proteasomal complexes attached to the plasma membrane have been described as nonconventional protein secretion systems. Once the proteins are degraded by these kinds of proteasomes, they are released into the extracellular space which in turn can stimulate postsynaptic neurons via NMDA-type receptors (involved in memory and learning). In addition, they are able to activate signals mediated by calcium [21]. Also, the application of proteasome inhibitors like MG132 induces a fast and several-fold increase in the frequency of spontaneous postsynaptic currents at excitatory and inhibitory synapses, which is independent of the accumulation of ubiquitylated proteins and specific by modulation of presynaptic neurons increasing the neurotransmitter release [24]. Furthermore, the inhibition of the proteasome system induces cell death in several cell types [22]. In neurons, the inhibition of proteasome has been shown to diminish the increase of cytosolic calcium that precedes programmed cell death, for example, before the activation of caspase-3 [22]. The progress of programmed cell death is very complex and depends on an orchestrated activation of proteins where calcium plays an important role. Experiments in primary cultures of neurons show that the activation of either a voltage-gated calcium channel or exchanger Na^+^-Ca^2+^ in the plasma membrane during the initial steps in cell death attenuates the damage via increase of cytosolic Ca^2+^. The inhibition of proteasome blocks this mechanism by reducing the increase of cytosolic calcium mediated by voltage-gated calcium channels [22]. Proteasome is also involved in other neural plasticity events like axonal growth, axonal guidance, and dendritic branching [23]. Failure of the proteostasis network may thus impede directly or indirectly the plasticity of neurons, by favoring the accumulation of aberrant proteins or modulating excitability, synapses, and growth.

Neurodegenerative diseases, which involve degradation of axons, loss of synapses, impairment of synaptic plasticity, and death of neurons, are one of the most enigmatic problems in medicine. Knowledge regarding these diseases has evolved from phenomenology description to mechanistic analysis, the hallmark being the aggregation and deposition of misfolded proteins [19]. AD, PD, and HD are today characterized by disrupted proteostasis, due to decreased function of the UPS, the accumulation of ubiquitylated proteins, and their aggregation, causing progressive neuronal dysfunction and death. Although ubiquitylated proteins can be localized in different brain areas, their high accumulation seems to be a common mechanism in all the diseases abovementioned and the UPS has been involved as primary or secondary cause. Mutations in genes encoding for UPS proteins [25] have also been associated with the development of hereditary forms of neurodegenerative diseases. Recent evidence shows that A*β* peptides, *α*-synuclein, and mutant huntingtin protein, which are at the origin of the three most important neurodegenerative diseases, share a specific oligomeric conformation that impairs proteasome function. According to this study, the shared three-dimensional structure allows these oligomers to potently inhibit 20S and 26S proteasome gate opening, thus drastically reducing its function. This effect blocks the degradation of proteins favoring its abnormal accumulation [26]. In neurodegenerative disease, as previously described in prion diseases [27], the misfolding protein acts as a template and interacts directly with the native protein and converts the latter into a misfolded replicate. This is the process that aberrant proteins use to recruit and propagate intracellularly the misfolding protein [28]. This seeded aggregation mechanism is employed by A*β* peptide, *α*-synuclein, and tau protein [28]. The accumulation of these proteins impairs the normal neuronal functions by altering the synaptic transmission [29] and causing cell death.

Besides neuronal dysfunction due to the accumulation of proteins, neurodegenerative disease could change the total protein expression. In particular in AD, a novel approach investigating postmortem the frontal cortex of sporadic AD patients using an integrated method of mass spectrometry-based quantitative proteomics revealed several clusters of modification of protein expression [30]. Using this method, the authors found 487 differentially expressed proteins with significantly altered levels. From this pool of proteins, 262 were upregulated while 225 were downregulated. In general terms, several functions in AD are altered which include proteostasis, RNA homeostasis, immune response, neuroinflammation, synaptic transmission, vesicular transport, cell signaling, cellular metabolism, lipid homeostasis, mitochondrial dynamics and function, cytoskeleton organization, and myelin-axon interactions. The identification of a wide spectrum of protein alterations strengthens the multifactorial and complex etiology of neurodegenerative disease and how the accumulation of altered proteins could alter completely the homeostasis of protein expression [30]. In the same line, AD proteomic applications indicate that the progression of the disease worsens several processes as energy production, signal transduction, synaptic plasticity, proteasome function, cellular morphology, and cell cycle [31].

In addition to protein misfolding and impaired proteostasis, neurodegenerative diseases are linked to imbalance of mitochondrial fission and fusion associated with an increase in oxidative stress. The association of mutant aberrant proteins with mitochondrial membrane has been reported to cause mitochondrial fragmentation, leading to mitochondrial dysfunction with concomitant production and liberation of reactive oxygen species. It is believed that this response would promote mitochondrial clearance by the cellular autophagic machinery via a process termed mitophagy [32], although the excess of activation of mitophagy could contribute to long-term neuronal degeneration [32]. This phenomenon is illustrated by PD where the abnormally degraded ubiquitylated proteins and *α*-synuclein often bind to mitochondrial membrane inducing mitochondrial dysfunction [33, 34].

Proteostasis is not limited to the cytoplasm only; it may occur in other cellular compartments. The most prominent are mitochondria and endoplasmic reticulum (ER), both organelles sharing multiple functions as calcium storage and lipid metabolism [35]. ER is considered the major site of cellular protein synthesis. One-third of the human proteome is synthesized in the ER, consisting in secreted proteins, integral membrane proteins, and functional proteins that connect the activity of ER and other organelles such as mitochondria [36].

*C. elegans*, *Drosophila*, and mammals, for instance, exhibit a mitochondrial unfolded protein response (mtUPR) against proteotoxic stress. This response could be activated by a wide range of noxious stimuli like depletion of mtDNA, impairment of mitochondrial chaperones or proteases, high concentration of ROS, or expression of misfolded proteins [37]. In general terms, this reaction consists in upregulating target genes that include organelle-specific chaperones and proteases to avoid the accumulation of toxic proteins [37]. Notably, this stress response is conserved in a cell culture model of HD, suggesting a general mechanism against stress [37].

Additional mechanisms may contribute to coordinated protein degradation between mitochondria and cytoplasm. The proteasome has been implied in the extraction and degradation of misfolded proteins of the mitochondrial outer membrane [38, 39]. In addition, it is possible that aggregates of cytosolic proteins can be sent to the mitochondria for their degradation by mitochondrial proteases [40]. Although this phenomenon remains incompletely understood, it is possible that the degradation system integrates the different cellular compartments to avoid protein aggregation not only in the cytoplasm but also in vital organelles such as endoplasmic reticulum and mitochondria, which can be altered by the aging process and neurodegenerative diseases. We present here below some examples of proteins involved in prevalent diseases and their particular role in neuron degeneration.

#### 1.1.1. Tau Protein

Tau protein is abundant in the central nervous system, and its main physiological function is to stabilize the cytoskeleton through binding to microtubules [41, 42]. Recent information indicates that tau protein is involved in several other processes such as synaptic plasticity and memory. A knockout mouse model for tau (Mapt^−/−^) evidenced aging-dependent short-term memory deficits, synaptic plasticity flaws, and impairment in long-term potentiation [43]. Some posttranslational modifications in tau protein such as phosphorylation, glycosylation, and ubiquitylation have been associated with neuropathologies. At the cellular level, Pick disease—a frontotemporal dementia that initiates with personality changes—is characterized by a large aggregation of hyperphosphorylated tau proteins that leads to production of Pick bodies [44, 45]. On the other hand, gliofibrillary tangles that characterize AD are composed of hyperphosphorylated tau proteins confined mainly to the entorhinal cortex [46, 47]. The accumulation of hyperphosphorylated tau is due to defective proteasomal degradation that may contribute to the build-up of tangles. In addition to phosphorylation, tau is also acetylated, and this modification impairs the proteasomal degradation and enhances the accumulation of tau. Together with A*β*-peptides, tau declines cognitive function, memory, and synaptic plasticity. These adverse effects produced by the combination of tau and A*β* can be prevented through the ablation of tau expression, leading to the hypothesis that tau is required for A*β*-induced synaptic dysfunction and memory deficits [43].

#### 1.1.2. *β*-Amyloid

*β*-Amyloid is also involved in AD. This protein is formed from amyloid precursor protein, which is processed by *α*-, *β*-, and *γ*-secretase [48]. While the form A*β*_40_ is the most common and soluble one, the more hydrophobic form A*β*_42_ is considered the most amyloidogenic and, therefore, predominant component of senile plaques [49]. In fact, a great accumulation of senile plaques is associated with UPS dysfunction with consequent synaptic dysfunction and neuronal loss in cortical and subcortical regions, leading to cognitive impairment, memory loss, and motor disturbances [49].

#### 1.1.3. *α*-Synuclein and PARK2

*α*-Synuclein is a soluble protein of 140 amino acids, which is abundant in neurons, and especially concentrated in presynaptic terminals [50]. This chaperone protein plays an important role in mediating protein-protein and protein-lipid interactions [51]. A mutated form of *α*-synuclein in patients with PD has been described [52], and again, the UPS is the main perturbed system favoring the accumulation of this protein. Selective inactivation of 26S proteasomes in substantia nigra dopaminergic neurons in a conditional knockout mouse model results in neurodegeneration and ubiquitin-positive aggregates resembling Lewy bodies (accumulation of *α*-synuclein). At a cognitive level, *α*-synuclein overexpression would induce a progressive loss of emotional memory secondary to mesolimbic dopaminergic dysfunction [53].

Parkin protein, now known as parkin RBR E3 ubiquitin-protein ligase (PARK2) [54], is part of the complex E3 ubiquitin ligase, necessary for the action of the ubiquitin-proteasome system. Parkin mutations have been associated with a familial form of early-onset PD [55, 56]. Interestingly, patients with PD with parkin mutations lack Lewy bodies, suggesting that parkin may be required for the formation and ubiquitination of these protein aggregates. Parkin has a role in neuroprotection by activating the PI3K-Akt pathway and also by cleansing dysfunctional mitochondria. Without the quality control of parkin, an increase in the number of dysfunctional mitochondria would lead to cell death. The dual-role context dependence of parkin should be better studied to understand neuronal physiology.

### 1.2. Coordinated Mitochondrial-Endoplasmic Reticulum Function Decline May Be Rescued by TRPV1 Control

The etiology of cognitive decline that occurs with aging is poorly understood; however, it is known that mitochondria are involved in this phenomenon [57]. Altered mitochondrial proteostasis and unfolded protein response could impede mitochondrial fusion and fission processes that normally reduce cell damage [14]. Disruptions of protein folding have also been associated to neurodegenerative disease with accumulation of misfolded proteins in the ER lumen, causing ER stress [35]. Several reports of increase in hyperphosphorylated tau protein in conjunction with stress markers in the ER in postmortem brain samples support this idea [58]. The “calcium hypothesis of brain aging and AD” intends to explain these findings. According to this hypothesis, A*β* would induce the ER to leach calcium that would be consequently taken by the mitochondria [59]. The calcium buffering mediated by mitochondria would induce overload of the ion in the mitochondrial matrix, reactive oxygen species production, and eventually, activation of programs of neuronal death [60, 61].

It should be noted that mammalian aging reduces pain perception associated with tissue damage by targeting the evolutionary conserved transient receptor potential cation channel subfamily V member 1 (TRPV1) that deploys a still unclear molecular mechanism for mitochondrial rescue [62]. TRPV1 mutations delay onset of age-related cognitive decline, maybe through SIRT1-dependent metabolic adaptation, which improves mitochondrial function and enhances several cellular antioxidant mechanisms [63]. The SIRT1 longevity factor is a deacetylase that plays a cytoprotective role in cellular response to stress. It is known that SIRT1 can modulate the heat shock response by deacetylation of the transcription factor HSF1, which triggers the production of molecular chaperones, promoting proteostasis and cellular viability [64]. In that sense, targeting mitochondrial proteostatic mechanisms, the natural TRPV1 agonist and antioxidant combined treatment synergistically would decrease glutamate toxicity, reactive oxygen species generation, and apoptotic neuronal death, offering a promising therapeutic approach to neurodegenerative disorders [65]. Activation of TRPV1 by capsaicin restores SIRT1 and suppresses NF-*κ*B signaling recovering tissue damage generated by plaques of atheroma [66]. In addition, leptin is able to reduce brain infarct volume and improve functional outcome after stroke via increased expression of TRPV1 and SIRT-1, restoring mitochondrial function and avoiding apoptosis [67].

### 1.3. Targeting Mitochondrial Dysfunction in Neuropsychiatric Disorders: The Case of Schizophrenia

As stated above, mitochondria have a prominent role in proteostasis [14, 68]. Mitochondria by themselves are responsible for producing cellular energy through the oxidative phosphorylation system, managing calcium buffering, generating reactive oxygen species, and storing regulators related to apoptosis. These functions are physiologically relevant due to the energetically expensive neuronal activities that lead to successful synaptic plasticity or cell death [69]. Many findings point out that mitochondrial function abnormalities are essential components of the underlying neurobiology of a number of neuropsychiatric conditions, including schizophrenia.

### 1.4. The Role of DISC1

Disrupted in schizophrenia 1 (DISC1) is a scaffold protein involved in the regulation of neuronal proliferation, differentiation, migration, and cytoskeletal modulation [70] which has been extensively linked to schizophrenia and other major mental illnesses [71–73]. Although it is expressed most highly during fetal neurogenesis and in the adult hippocampus, DISC1 is expressed in different brain regions [74] and in other tissues as well [75]. DISC1 interactions with proteins of the dopaminergic system, such as fasciculation and elongation protein zeta 1, phosphodiesterase 4D9 and phosphodiesterase 4B, serine/threonine protein kinase Akt, and glycogen synthase kinase-3, have been studied due to their therapeutic potential [76, 77].

Unregulated expression of DISC1 and aberrant multimerization of DISC1-producing insoluble aggregates that are dysfunctional are associated with chronic neuropsychiatric diseases [75, 77]. Insoluble oligomers of DISC1 have indeed been found in postmortem brain samples of patients with schizophrenia [78]. The DISC1 mutant gene resulting from balanced translocation t(1;11)(q42;q14.3) was first identified in a Scottish lineage, and then it was found in other families, all of them with a history of schizophrenia among other mental disorders [79, 80]. In a recent systematic review, it was concluded that DISC1 would have a role in the regulation of dopaminergic function, installing dopaminergic dysregulation as a possible explanation for the higher rate of schizophrenia observed in patients with the DISC1 variant [77].

Inheritance of maternal mitochondrial DNA variants might be associated with the high prevalence of the disorder in relatives of schizophrenic patients [81]. Thus, Rollins et al. [82] verified that the synonymous base pair substitutions in the coding regions of the mitochondrial DNA genome in the dorsolateral prefrontal cortex of schizophrenics were increased by 22% compared to controls. Mostly found in mitochondria [83], DISC1 has been demonstrated to participate in neurite outgrowth, neurogenesis, neuronal migration, intracellular cAMP signaling, and many other neuronal processes [69]. Mitochondrial overexpressed truncated DISC1 isoforms may determine abnormal mitochondrial morphology, and depletion of DISC1 causes deficiencies in important mitochondrial enzyme activities and interferes mitochondrial trafficking throughout the axons [84]. Hence, the processes mediated by DISC1 in mitochondrial dynamics are necessary for neural development and dendritic branching [85]. Recent findings have shown that DISC1 plays a central role in mitochondrial function in association to mitofilin, a single-span mitochondrial inner membrane protein that is crucial for regulating mitochondrial cristae morphology and for preservation of mitochondrial DNA [69, 86]. DISC1 deficiencies are also linked with mitochondrial dysfunction such as decreased NADH dehydrogenase activity in the electron transport chain, reduced ATP contents, impaired mitochondrial calcium dynamics, and diminished activity of monoamine oxidase, which can be related to the loss of mitofilin stability as well as mitochondrial morphological abnormalities. Particularly, downregulation of monoamine oxidase activity is of utmost interest due to its link with the mesolimbic hyperdopaminergic tone, probably responsible for positive psychotic symptoms. Consequently, monoamine oxidase activity in DISC1-deficient neurons might indeed be a key element in hyperdopaminergic theory [69, 86].

In a critical and recent study of Park et al. [87], DISC1 deficiency is shown to elicit a hyperactivation in endoplasmic reticulum-mitochondrial Ca^2+^ transfer—through the mitochondrial associated endoplasmic reticulum membrane—triggered by oxidative stress and excessive glucocorticoids, causing abnormal mitochondrial Ca^2+^ storage. This process finally triggers an overproduction of ROS mediated by a disruption in mitochondrial membrane potential [87]. The authors concluded that DISC1 modulates neuronal stress response through ER-mitochondrial Ca^2+^ transfer. Thus, DISC1 association with cognitive and emotional deficits implies dysregulation of Ca^2+^ flux between ER and mitochondria through mitochondrion-associated membrane proteins and the consequent loss of proteostasis as a common mechanism shared by aging, as well as neurodegenerative and psychiatric diseases.

In other animal model explorations, DISC1 has been implicated in hypothalamic-pituitary-adrenal dysregulations [88, 89]. Specifically, in a mouse model it has been demonstrated that environmental stressors combined with an appropriate genetic risk can trigger, for example, neurochemical projections originating from the ventral tegmental area and behavioral changes induced by DISC1 expression [89]. Interestingly, these findings have allowed formulating the hypothesis that environmental stressors during childhood and adolescence could exert epigenetic control over the dopaminergic pathways and, therefore, set mental illnesses as schizophrenia.

#### 1.4.1. Dendritic Spines and Mitochondrial Hypoplasia

Different studies indicate that the mitochondrial network displays important transcriptome alterations in layer III pyramidal cells in schizophrenics, supporting a molecular link between mitochondrial dysfunction and the important decrease in dendritic spine density observed in these neurons [90]. Mitochondria regulate dendritic spine morphogenesis and plasticity but are also involved in the negative regulation of dendritic branching during development. Overall, evidence intrinsically links mitochondrial copy number, localization, and function with dendritic spine morphology and synaptic transmission [91]. In this context, the most frequently found protein in postsynaptic density is PSD-95, a scaffolding protein which belongs to the kinase family. It is implied in excitatory synapses and plays a key role in synaptic plasticity through dendritic spine morphogenesis and long-term potentiation and long-term depression. Postmortem studies carried out in brains of schizophrenic patients have demonstrated a significant decrease in PSD-95 mRNA levels in specific areas as dorsolateral and dorsomedial prefrontal cortices [92]. This may be related to anomalous spine dynamics observed in neurodevelopmental and neuropsychiatric disorders, for example, schizophrenia and autism spectrum disorders [93]. Different explorations in patients with schizophrenia have found decreased numbers of mitochondria in presynaptic buttons in dopaminergic neurons of the substantia nigra [94]. Moreover, a reduction in the number of mitochondria in axons of drug-naïve schizophrenics has also been verified, but not in patients using antipsychotic drugs [95]. Findings also exhibit significant decreases in the mitochondrial density of oligodendroglial cells in the caudate nucleus and prefrontal areas in patients, particularly those with prominent negative symptoms [96].

#### 1.4.2. Inflammation

Neuroprogression, a stage-related phenomenon of neurodegeneration and decline in neuronal plasticity and neurogenesis that has been employed as a research paradigm in schizophrenia, has demonstrated to be significantly influenced by neuroinflammation due to a synergistic effect with mitochondrial dysfunction and neuroprogressive immunoinflammatory, oxidative, and nitrosative stress pathways, activating a vicious cycle that conduces to neuronal death [97, 98]. Novel therapeutic strategies could focus on improving mitochondrial function, through promoting an endogenous antioxidant defense system and antioxidant treatment to compensate mitochondrial injury and increase the mitochondrial respiration rate [97].

Another potential therapeutic target regarding mitochondrial functioning is the translocator protein, located in the outer mitochondrial membrane of steroid-synthesizing nervous cells. It is involved in the permeability to water and small substances at the junction of the inner and outer membranes. Since it is linked with apoptosis and upregulated in some neurodegenerative diseases, this protein has been proposed as an inflammation biomarker and is currently being appraised in clinical trials of drug use [99].

#### 1.4.3. Electron Transport Chain

Diverse neuroimaging studies have demonstrated an altered metabolism expressed as changes in ATP in different brain regions of schizophrenic patients [5]. The severity of negative symptoms and the neuropsychological performance would be correlated with ATP levels [100]. These results point out a dysfunction of brain mitochondrial oxidative phosphorylation, related intrinsically with processes as pre- and postsynaptic action potentials, neurotransmitter release, and postsynaptic currents [101, 102]. Specifically, the expression of multiple complex I subunits of the electron transport chain, such as NDUFV1, NDUFV2, and NDUFS1, is significantly altered in the prefrontal cortex, striatum, hippocampus, and parietooccipital cortex of schizophrenics [102, 103]. In fact, the NDUFV2 gene has been included as a high-risk gene for schizophrenia [104]. In this regard, a study conducted by Robicsek et al. [105] corroborated the impairments in maturation and differentiation into dopaminergic and glutamatergic neurons of schizophrenic-derived pluripotent stem cells, alongside a reduction in complex I-driven respiration, dissipation in mitochondrial membrane potential, altered mitochondrial network structure and connectivity, and aberrant expression degrees of NDUFV1, NDUFV2, and NDUFS1. Some interactions have also been proposed between oxidative phosphorylation and intramitochondrial calcium as complex I, complex II, and complex IV alterations are linked with abnormalities in calcium signaling [106].

With regards to pharmacotherapy, self-defeating findings indicate that typical and atypical antipsychotic drugs would inhibit complex I activity and complex I-driven respiration in isolated mitochondria and in intact neurons [102]. Comparable to these effects, dopamine also affects mitochondrial activity in neuronal cultures by diminishing complex I function and ATP synthesis. These findings could be related to the mitochondrial dopamine uptake, provoking a dose-dependent inhibition of complex I functioning [107]. Both antipsychotics and dopamine inhibit complex I activity, although they interact with the complex at different sites: dopamine interacts with the hydrophilic matrix-penetrating arm and antipsychotics with the hydrophobic inner membrane-embedded arm of the complex. While therapeutic effects of these drugs are due to their antagonism of the D2 receptor, side effects of antipsychotics might be explained by this drug-mitochondria interaction. Besides, dopamine and antipsychotic drugs may interact independently with mitochondria, participating in a compensatory phenomenon with the aim of overcoming mitochondrial dysfunction [102].

## 2. Conclusions

Our results show that a dysfunction of the proteostasis system is implicated in the etiology of a series of highly prevalent psychiatric and neurodegenerative processes such as PD, dementia, and schizophrenia, among others [12]. Indeed, proteostasis alterations in the prefrontal cortex implicate proteome instability and accumulation of misfolded proteins [45, 47, 49, 76] that could lead to detrimental behavioral and emotional functions in neuropsychiatric disorders [108]. Furthermore, altered mitochondrial dynamics, proteostasis, and mitochondrial unfolded protein response could impede mitochondrial fusion and fission, processes that normally reduce cell damage [14]. This may be related to the decline in prefrontal cortex performances observed during aging [109]. Mitochondrial alterations, specifically on its genetic bases [69, 86, 110], regulatory role in dendritic development [90, 91] and neuroinflammation [97, 98], could be the underlying phenomena of psychiatric disorders as schizophrenia. In the context of the neuronal relevance of mitochondrial functions [69], we hypothesize that it is possible to delay onset of age-related cognitive decline through metabolic SIRT1-dependent adaptation and improvement of mitochondrial function mediated by TRPV1 control. Thereby, TRPV1 modulation of the mitochondrial proteostasis mechanism could be used to design drug strategies against neural-dependent conditions, such as detrimental cognitive performance. Finally, we expect that with the increasing evidence about proteostasis in psychiatric and neurodegenerative disorders, new therapeutic alternatives will emerge.
